# Identification and integrated analysis of glyphosate stress-responsive microRNAs, lncRNAs, and mRNAs in rice using genome-wide high-throughput sequencing

**DOI:** 10.1186/s12864-020-6637-6

**Published:** 2020-03-17

**Authors:** Rongrong Zhai, Shenghai Ye, Guofu Zhu, Yanting Lu, Jing Ye, Faming Yu, Qiren Chu, Xiaoming Zhang

**Affiliations:** 1 Institute of Crop and Nuclear Technology Utilization, Zhejiang Academy of Agricultural Sciences, 198, Shiqiao Road, Hangzhou, 310021 Zhejiang China; 2 RiceTec Inc, Alvin, TX 77511 USA

**Keywords:** Rice, Transcriptome sequencing, Degradome sequencing, Glyphosate, Long non-coding RNA

## Abstract

**Background:**

Glyphosate has become the most widely used herbicide in the world. Therefore, the development of new varieties of glyphosate-tolerant crops is a research focus of seed companies and researchers. The glyphosate stress-responsive genes were used for the development of genetically modified crops, while only the *EPSPS* gene has been used currently in the study on glyphosate-tolerance in rice. Therefore, it is essential and crucial to intensify the exploration of glyphosate stress-responsive genes, to not only acquire other glyphosate stress-responsive genes with clean intellectual property rights but also obtain non-transgenic glyphosate-tolerant rice varieties. This study is expected to elucidate the responses of miRNAs, lncRNAs, and mRNAs to glyphosate applications and the potential regulatory mechanisms in response to glyphosate stress in rice.

**Results:**

Leaves of the non-transgenic glyphosate-tolerant germplasm CA21 sprayed with 2 mg·ml^− 1^ glyphosate (GLY) and CA21 plants with no spray (CK) were collected for high-throughput sequencing analysis. A total of 1197 DEGs, 131 DELs, and 52 DEMs were identified in the GLY samples in relation to CK samples. Genes were significantly enriched for various biological processes involved in detoxification of plant response to stress. A total of 385 known miRNAs from 59 miRNA families and 94 novel miRNAs were identified. Degradome analysis led to the identification of 32 target genes, of which, the squamosa promoter-binding-like protein 12 (SPL12) was identified as a target of osa-miR156a_L + 1. The lncRNA-miRNA-mRNA regulatory network consisted of osa-miR156a_L + 1, two transcripts of *SPL12* (*LOC_Os06g49010.3* and *LOC_Os06g49010.5*), and 13 lncRNAs (e.g., MSTRG.244.1 and MSTRG.16577.1).

**Conclusion:**

Large-scale expression changes in coding and noncoding RNA were observed in rice mainly due to its response to glyphosate. *SPL12*, osa-miR156, and lncRNAs (e.g., MSTRG.244.1 and MSTRG.16577.1) could be a novel ceRNA mechanism in response to glyphosate in rice by regulating transcription and metal ions binding. These findings provide a theoretical basis for breeding glyphosate-tolerant rice varieties and for further research on the biogenesis of glyphosate- tolerance in rice.

## Highlights

1. A total of 385 known miRNAs of 59 miRNA families and 94 novel miRNAs were identified.
2. In all, 1197 DEGs, 131 DELs, and 52 DEMs were identified in GLY samples vs. CK.
3. The DEGs were enriched in biological processes involving in detoxification of plant response to stress.
4. *SPL12* was identified to be a target of osa-miR156 by degradome sequencing analysis.
5. The ceRNA network contained *SPL12*, osa-miR156 and several lncRNAs, which could be a novel glyphosate stress-responsive ceRNA mechanism in rice.

## Background

Rice (*Oryza sativa* L.) is one of the most important food crops for a large segment of the world population, and plays a crucial role in agricultural production [1]. Approximately 90% of the world’s rice is produced and consumed in Asia [2], and its production and quality are directly related to people’s lives and national economic stability [3]. Nevertheless, increasing rice production can be challenging, owing to water scarcity, declining utilization of cultivated land, and other factors [4]. Removal of rice weeds is one of the problems that hinders large-scale production of rice, as the weeds not only compete with rice for soil, water, and fertilizer, but also may harbor and spread pests and diseases, which could reduce the yield [5]. Traditional weeding methods involve hoeing or hand-pulling, and are labor-intensive. In contrast, chemical herbicides are widely used to save time, labor and application costs [2].

Glyphosate, N-(phosphonomethyl) glycine, is a broad-spectrum, non-selective, and post-emergence herbicide, widely used in agricultural and non-agricultural lands to control annual/perennial weeds [6]. Glyphosate has become the most widely used herbicide in the world. Therefore, the development of new varieties of glyphosate-tolerant crops is a research focus of seed companies and researchers. Glyphosate is sprayed on the stem and leaves. It functions by inhibiting the 5-enolpyruvylshikimate-3-phosphate synthase (EPSPS) enzyme, which interrupts the synthesis of aromatic amino acid, and further leads to protein deficiency, leading to plant death [7]. The glyphosate stress-responsive genes used for the development of genetically modified crops include *EPSPS*, glyphosate acetyltransferase (*GAT*), and glyphosate oxidoreductase (*GOX*) [8], of which *EPSPS* is widely used in glyphosate-tolerant rice. Although successful transformation of several glyphosate stress-responsive genes such as *OsEPSPS* [9], *MdEPSPS* [10], *VvEPSPS* [11], *G2-aroA* [8], *G6* [7], *AroAJ.sp* [12], and *I. variabilis-EPSPS* [13], have been reported in rice, these transgenic offspring carry exogenous genes. Commercialization of transgenic varieties is greatly hindered, particularly, given the heightened biosafety concerns of transgenic breeding. The glyphosate-tolerant rice has been successfully created by the fixed-point replacement of two amino acids (T102I and P106S, TIPS) in the conserved region of the endogenous *EPSPS* gene in rice using CRISPR/Cas9 technology [14]. Nevertheless, only the *EPSPS* gene has been used currently in the study on glyphosate-tolerance in rice. Therefore, it is essential and crucial to intensify the exploration of glyphosate stress-responsive genes, to not only acquire other glyphosate stress-responsive genes with clean intellectual property rights but also obtain non-transgenic glyphosate-tolerant rice varieties.

Non-coding RNAs (ncRNA) refer to the RNAs that lack the ability to encode proteins, which were initially regarded as inessential transcriptional ‘noises’. Research advances have demonstrated the crucial regulatory roles of ncRNAs in various biological processes [15]. Small RNA (sRNA) are key riboregulators, implicated in genome stability, and adaptive responses through the regulation of gene expression by acting on RNA and DNA [16]. sRNA include three major categories, small interfering RNA (siRNA), Piwi-interacting RNA (piRNA) and microRNA (miRNA). miRNAs are 22 nt long endogenous ncRNAs in plants, which mediate post-transcriptional gene expression [17]. In plants, miRNAs are implicated in multiple biological processes, including developmental regulation, stress and hormone response, and plant flowering [18–20]. For example, Yang et al. revealed that over-expression of osa-miR319 caused the leaves to become wider probably by increasing the longitudinal small veins, and improved cold-tolerance in rice by down-regulating their target genes *OsPCF5/OsPCF8*. The ncRNAs with length > 200 nucleotides are regarded as long ncRNAs (lncRNAs), which are always expressed at low levels and have short conserved sequences [21]. LncRNAs are found to be involved in post-transcriptional gene regulation and chromatin modifications [22, 23]. Despite the recent recognition of the key roles of lncRNAs, plant lncRNAs are shown to be involved in photomorphogenesis, auxin transport, and flowering [24, 25]. For instance, lncRNAs COLDAIR and COOLAIR are shown to mediate flowering time probably by regulating the FLOWERING LOCUS C (*FLC*) in *Arabidopsis* [26, 27]. However, there is no report on the preliminary elucidation of the mechanism in response to glyphosate stress using an integrated analysis using high-throughput sequencing technology.

In a previous study, the non-transgenic glyphosate-tolerant germplasm CA21 with independent intellectual property rights has been created using chemical mutagenesis by irradiation + ethyl methane sulfonate (EMS) and combined with traditional hybrid breeding methods [28]. Unlike transgenic rice with the glyphosate stress-responsive gene, the glyphosate-tolerant germplasm CA21 obtained by mutagenesis has no risks of field release and has broad application prospects in landscaping. It will also create conditions favoring the opening of the international rice market by mastering the key technologies of glyphosate-tolerant rice varieties.

In this study, we first constructed cDNA libraries, sRNA libraries, and degradome libraries from the leaves of glyphosate-tolerant rice CA21. Through high-throughput sequencing analysis, a systematic and integrated analysis of the potential lncRNA-miRNA-mRNA regulatory network was performed in leaves of glyphosate-tolerant rice. This study is expected to elucidate the responses of miRNAs, lncRNAs, and mRNAs to glyphosate applications and the potential regulatory mechanisms in response to glyphosate stress in rice.

## Results

### Physiological responses to glyphosate

**Fig. 1:**
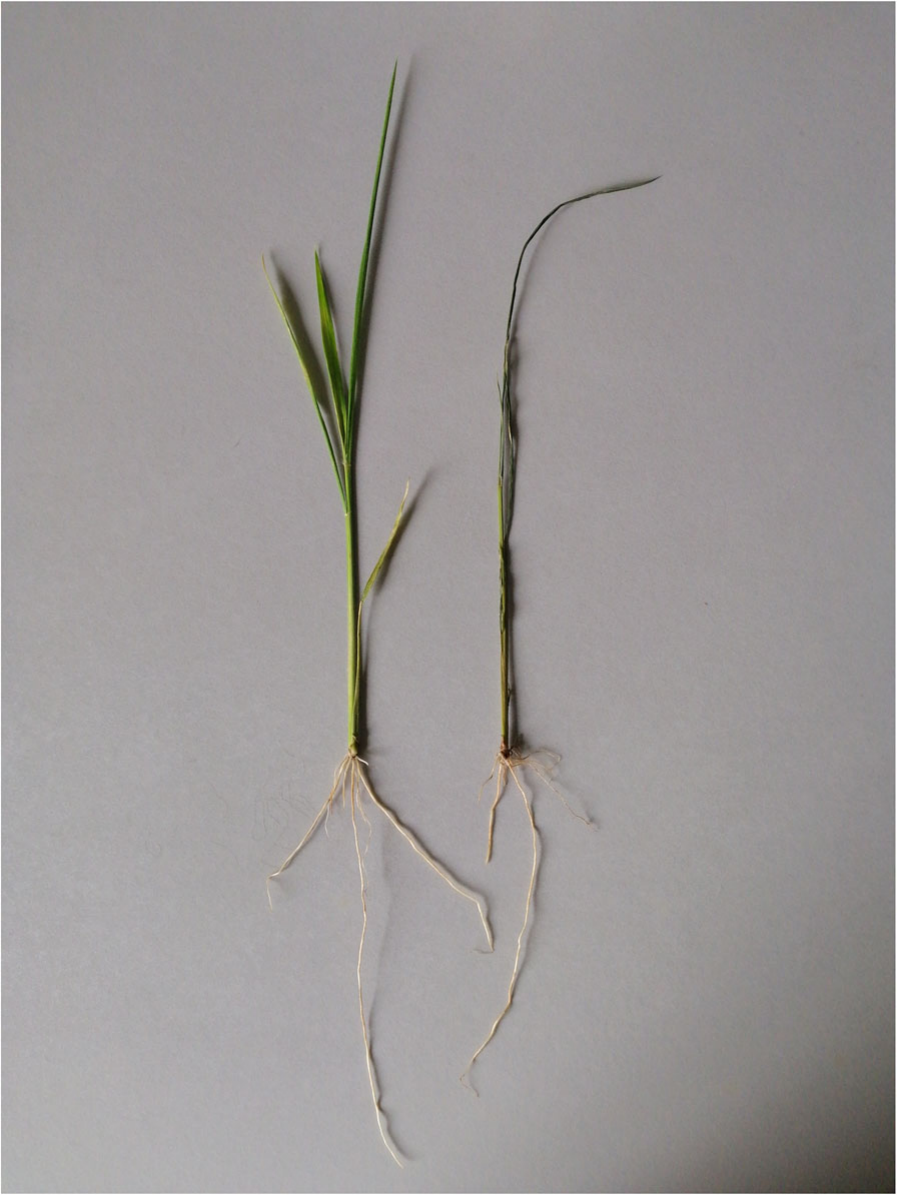
Growth status of CA21 and P1003 rice plants on the third day after glyphosate treatment. CA21, glyphosate-resistant rice grows normally while P1003, glyphosate-sensitive rice displays a curled heart leaf and death

The glyphosate-tolerant rice CA21 grew normally, while the heart leaf of the glyphosate-sensitive rice P1003 curled on the third day after glyphosate treatment (Fig. 1). Shikimic acid, MDA and GST levels were found to be increased in CA21 and P1003 treated with glyphosate, when compared to that in the control plants (Table 1). Notably, the shikimic acid content increased significantly by 42.56% in P1003, which increased only by 10.27% in CA21 plants. Similarly, the MDA content in P1003 increased by up to 58.87%, which only increased by 12.59% in CA21. In contrast, the GST content of CA21 increased remarkably by up to 233.33%, while these only doubled in P1003. Hence, we speculate that the tolerance of CA21 to glyphosate is likely achieved by increasing the detoxification activity of GSTs.

**Table 1:** The results of the determination of physiological indicators

Samples (concentration of glyphosate)	Shikimic acid (mg/g)	MDA (nmol/g)	GSTs (U/g)
GLY (2 mg/ml)	11.38 ± 0.14^a^	17.35 ± 0.82^a^	0.01 ± 0.00^a^
CK (0 mg/ml)	10.32 ± 0.01^b^	15.41 ± 0.16^b^	0.003 ± 0.00^b^
P1003 (2 mg/ml)	14.57 ± 0.03^a^	24.26 ± 0.72^a^	0.004 ± 0.00^a^
P1003 (0 mg/ml)	10.22 ± 0.11^b^	15.27 ± 0.16^b^	0.002 ± 0.00^b^

The superscript “a” and “b” represent that there is a statistic difference of *P* < 0.05 between samples with or without glyphosate treatment for the contents of each physiological indicator

### mRNA expression profile analysis

**Fig. 2:**
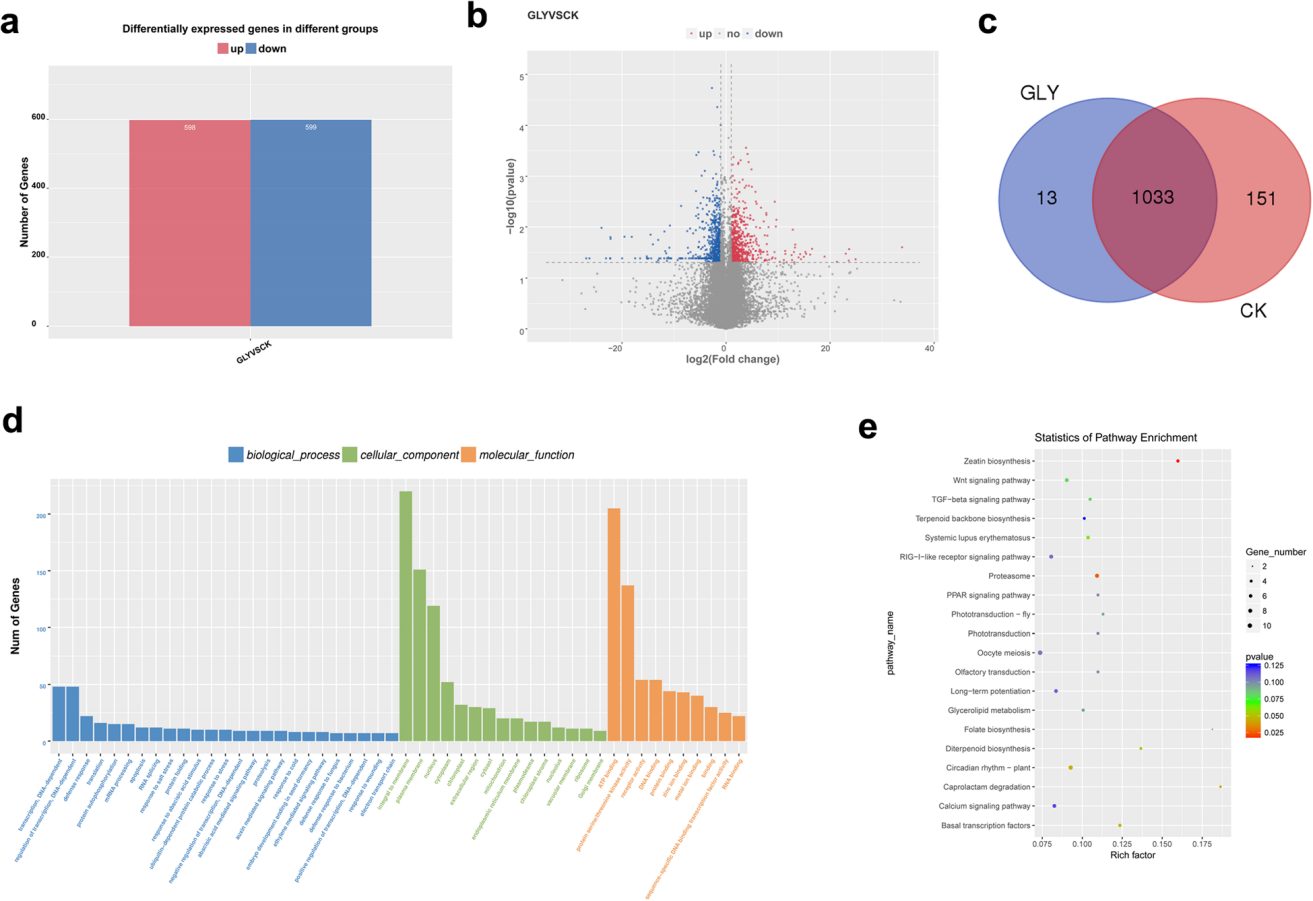
Identification and characterization of differentially expressed genes (DEGs) between glyphosate-resistant rice (GLY) and glyphosate-sensitive rice (CK) plants. **a** the number of up- and down-regulated DEGs. **b** Volcano Plot of DEGs. **c** Venn diagram of DEGs between GLY and CK samples. **d** part of the significantly enriched GO terms; **e**, part of the significantly enriched KEGG pathways

High-throughput sequencing generated 692,668,924 raw reads in the leaf samples of CA21 plants that were sprayed with 2 mg ml^− 1^ glyphosate (GLY) and CA21 devoid of any treatment (CK). Over 96% of the sequences were considered to be valid reads (Table 2). The valid reads were mapped to the Rice Genome utilizing TopHat, and the uniquely mapped reads ranged from 68.96–75.70% (Supplementary Table S1). In addition, they were mainly mapped to the exons (approximately 93%), followed by introns (approximately 4%) and intergenic regions (approximately 2%) (Supplementary Figure S1). In all, the expression of approximately 26,047 genes in CK (25,984/ 26,196/, and 25,963 genes of CK1, CK2, and CK3 samples) and 26,834 genes (26,978/ 26,713 /26,812 genes of GLY1, GLY2, and GLY3 samples) in GLY were detected (Supplementary Table S2). The FPKM value for each gene was calculated to evaluate the expression level of the gene, and 1197 genes with clearly differential-expression were found in the leaves of GLY versus CK, including 598 up-regulated genes and 599 down-regulated genes (Fig. 2a and b). As shown in the Venn diagram of the differentially expressed genes (DEGs), 13 genes were specific to GLY samples, 151 genes were specific to CK samples, and 1033 genes were common to both GLY and CK samples (Fig. 2c).

**Table 2:** Statistical data of the reads for six cDNA libraries

Sample	Raw Data		Valid Data		Valid Ratio (reads)	Q20%	Q30%	GC content%
	Reads	Bases	Reads	Bases				
CK1	120,000,000	18.00G	117,231,082	17.58G	97.69	99.75	95.32	46
CK2	120,000,000	18.00G	117,257,130	17.59G	97.71	99.77	95.59	47
CK3	120,000,000	18.00G	117,181,868	17.58G	97.65	99.78	95.79	47
GLY1	117,206,668	17.58G	113,460,552	17.02G	96.80	99.82	96.03	47.50
GLY2	108,982,164	16.35G	106,639,642	16.00G	97.85	99.74	95.21	47
GLY3	106,480,092	15.97G	103,696,664	15.55G	97.39	99.59	95.10	47

To further explore the functions of these DEGs, functional enrichment analysis was conducted, including GO terms and KEGG pathways. As shown in Fig. 2d, there were three categories of the GO terms. The DEGs were significantly enriched (*P* < 0.05) in 55 GO_biological process (BP) terms, such as GO:0009691~cytokinin biosynthetic process, GO:0045892~negative regulation of transcription, DNA-dependent, 35 GO_molecular function (MF) terms, for example GO:0008121~ubiquinol-cytochrome-c reductase activity, and 13 GO_cellular component (CC) terms, for instance, GO:0009570~chloroplast stroma. In addition, the DEGs were found to be related to zeatin biosynthesis, proteasome, caprolactam degradation and other pathways (Fig. 2e). In addition, the GO-terms associated with plant response to stress, including, GO:0004601~peroxidase activity, GO:0006749~glutathione metabolic process, and GO:0043620~regulation of DNA-dependent transcription in response to stress, were also enriched.

### LncRNA expression profile analysis

**Fig. 3:**
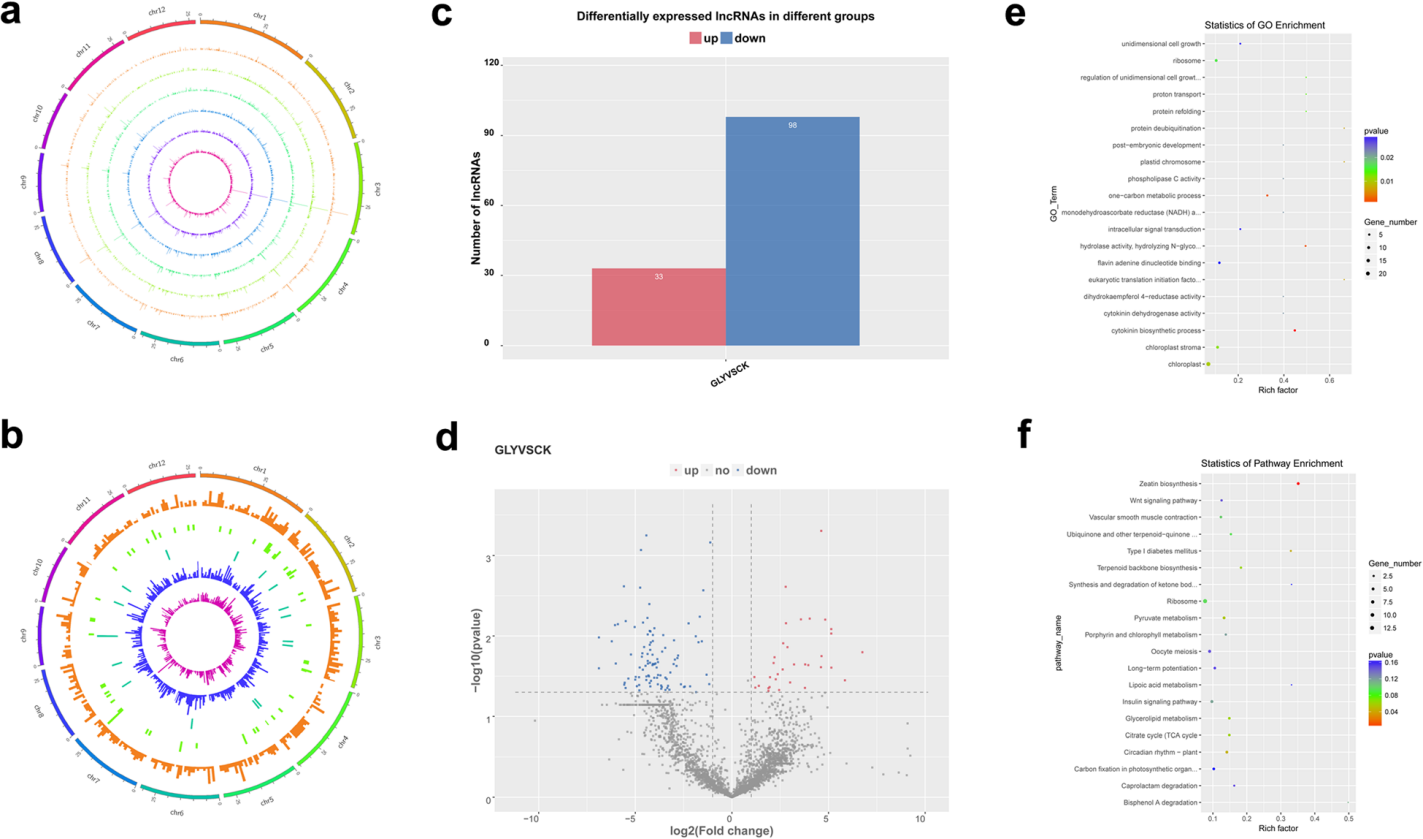
Identification and characterization of differentially expressed lncRNAs (DELs) between GLY and CK samples. **a** expression levels of lncRNA on 12 chromosomes of rice. Each circle represents one sample and corresponds to CK 1/2/3 and GLY 1/2/3 samples from the outer to inner. **b** the location of lncRNA types on the 12 chromosomes. Each circle represents a type of lncRNA and corresponds to “i”, “j”, “o”, “u”, and “x” from the outer to inner. “i”, transfrag falling entirely within a reference intron; “j”, potentially novel isoform; “o”, generic exonic overlap with a reference transcript; “u”, intergenic transcript; “x”, exonic overlap with a reference to the opposite strand. **c** the number of up-r and down-regulated DELs. **d** Volcano Plot of DELs. **e** part of the significantly enriched GO terms for the target genes of DELs. **f** part of the significantly enriched KEGG pathways for the target genes of DELs

The lncRNAs were identified as per the method described above. Briefly, the genes were assembled, annotated, and filtered based on its coding potential and length. Consequently, the remaining genes were regarded as lncRNAs. There were 1771, 1649, 1679, 1677, 1633 and 1631 novel lncRNAs identified from the six cDNA libraries, respectively (Supplementary Table S3). Altogether, 3691 novel lncRNAs were identified, of which 898 lncRNAs were specific to CK samples, and 771 lncRNAs were specific to GLY samples (Supplementary Table S4). These lncRNAs were found to be evenly distributed across the 12 chromosomes in rice (Fig. 3a). The types of lncRNAs were class_code “u” (intergenic transcript, 38–40.29%), class_code “x” (exonic overlap with reference on the opposite strand, 33.93–36.17%) and class_code “i” (transfrag falling entirely within a reference intron, 21.03–24.51%), followed by class_code “j” (potentially novel isoform, 2.62–3.25%) and class_code “o” (generic exonic overlap with a reference transcript, 0.62–0.85%), and these lncRNAs had no marked preference for genome locations (Supplementary Figure S2 and Fig. 3b). In addition, 131 lncRNAs were differentially expressed between GLY and CK samples, including 33 up-regulated and 98 down-regulated lncRNAs (Supplementary Table S4, Fig. 3c and d).

To further investigate the potential roles of the differentially expressed lncRNAs (DELs), we first predicted their target genes that were cis-regulated. The functions of lncRNAs were obtained by analyzing the GO terms and KEGG pathways of their target genes. A total of 45 DEGs were predicted to interact with 37 DELs (Supplementary Table S5), and they were found to be significantly enriched in 29 GO terms, including 13 GO_BP terms, for example, GO:0009691~cytokinin biosynthetic process; 5 GO_CC terms, for instance, GO:0009508~plastid chromosome; and 11 GO_MF terms, for example, GO:0016799~hydrolase activity, hydrolyzing N-glycosyl compounds (Fig. 3e). In addition, 70 KEGG pathways were significantly enriched, including zeatin biosynthesis and plant circadian rhythm (Fig. 3f).

### Overview of sRNA sequencing data and miRNA identification

**Fig. 4:**
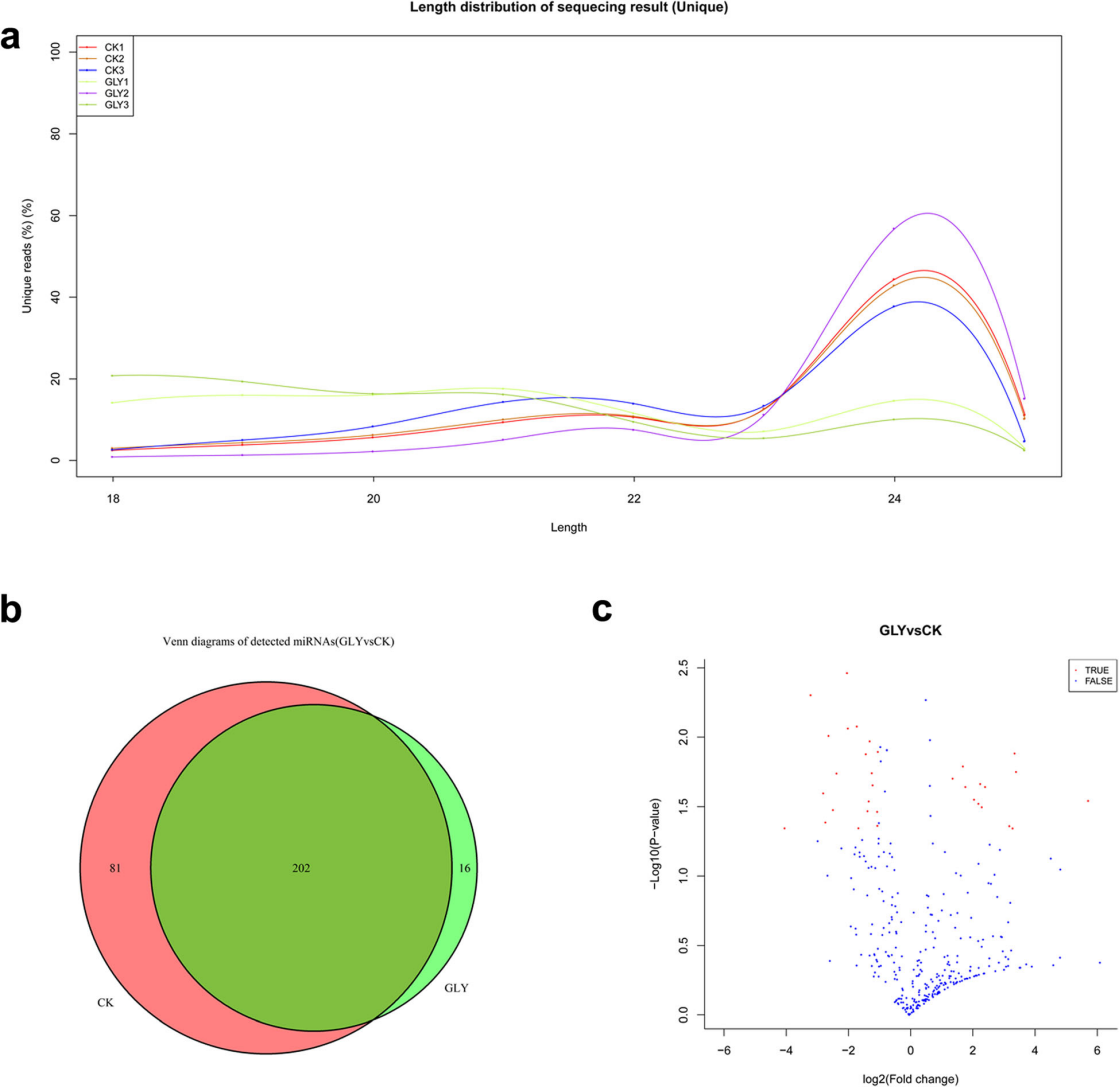
Identification and characterization of miRNAs in GLY and CK samples. **a** length distribution of the unique reads. **b** Venn diagram of differentially expressed miRNAs between GLY and CK samples. **c** Volcano Plot of differentially expressed miRNAs

To explore the glyphosate stress-responsive miRNAs in rice, six sRNA libraries were constructed from the leaves of GLY and CK plants and sequenced. High-throughput sequencing generated an average of 12,977,314 (13,774,024/ 13,774,024/ 11,305,213 for CK1/ CK2/ CK3) and 11,396,302 (10,234,674/ 14,038,269/ 9,915,965 for GLY1/ GLY2/ GLY3) raw reads in GLY and CK samples, respectively. After removing the adapter sequences, low complexity sequences and reads smaller than 18 nt, an average of 2,310,371 (2,453,175/ 2,405,519/ 2,072,418 for CK1/ CK2/ CK3) and 2,016,847 (1,828,494/ 2,749,254/ 1,472,793 for GLY1/ GLY2/ GLY3) unique reads were obtained (Table 3). The length distribution of the unique reads showed that majority of the sRNAs was 24 nt in length (Fig. 4a). The six sRNA libraries shared similar sRNA types and proportion, including rRNA (approximately 6%), tRNA (among 0.01–0.02%), snRNA (among 0.01–0.02%), snoRNA (approximately 0.04%), and other Rfam RNA (approximately 0.35%). The detailed information of each library was presented in Table 3.

**Table 3:** Summary of small RNA (sRNA) sequencing data

	lib	Raw reads	3ADT&length filter	Junk reads	Rfam	mRNA	Repeats	valid reads	rRNA	tRNA	snoRNA	snRNA	other Rfam RNA
CK1	Total	13,774,024	3,390,537	38,220	**1,054,000**	847,233	4749	8,495,423	863,173	139,387	5115	1723	44,602
	% of Total	100.00	24.62	0.28	**7.65**	6.15	0.03	61.68	6.27	1.01	0.04	0.01	0.32
	uniq	2,453,175	841,448	20,791	**18,859**	14,165	185	1,558,672	15,033	2264	332	159	1071
	% of uniq	100.00	34.30	0.85	**0.77**	0.58	0.01	63.54	0.11	0.02	0.00	0.00	0.01
CK2	Total	13,852,706	2,934,133	33,314	**1,258,384**	973,925	6008	8,708,898	1,083,866	115,231	4395	1803	53,089
	% of Total	100.00	21.18	0.24	**9.08**	7.03	0.04	62.87	7.82	0.83	0.03	0.01	0.38
	uniq	2,405,519	874,287	17,335	**19,294**	14,955	214	1,480,435	15,747	1975	291	149	1132
	% of uniq	100.00	36.35	0.72	**0.80**	0.62	0.01	61.54	0.11	0.01	0.00	0.00	0.01
CK3	Total	11,305,213	751,727	31,484	**861,890**	947,345	4370	8,761,939	698,077	121,230	4076	1822	36,685
	% of Total	100.00	6.65	0.28	**7.62**	8.38	0.04	77.50	6.17	1.07	0.04	0.02	0.32
	uniq	2,072,418	519,795	17,449	**18,638**	13,555	182	1,503,706	14,936	2174	299	140	1089
	% of uniq	100.00	25.08	0.84	**0.90**	0.65	0.01	72.56	0.13	0.02	0.00	0.00	0.01
GLY1	Total	10,234,674	3,616,701	19,106	**1,149,522**	967,205	17,295	4,557,991	961,780	124,212	6855	2549	54,126
	% of Total	100.00	35.34	0.19	**11.23**	9.45	0.17	44.53	9.40	1.21	0.07	0.02	0.53
	uniq	1,828,494	917,707	7845	**18,067**	13,035	282	872,751	14,296	2195	320	142	1114
	% of uniq	100.00	50.19	0.43	**0.99**	0.71	0.02	47.73	0.14	0.02	0.00	0.00	0.01
GLY2	Total	14,038,269	7,373,691	39,460	**857,843**	617,980	1416	5,187,783	722,433	94,555	3929	2256	34,670
	% of Total	100.00	52.53	0.28	**6.11**	4.40	0.01	36.95	5.15	0.67	0.03	0.02	0.25
	uniq	2,749,254	1,226,291	25,154	**14,288**	11,708	77	1,472,281	11,735	1340	291	145	777
	% of uniq	100.00	44.60	0.91	**0.52**	0.43	0.00	53.55	0.08	0.01	0.00	0.00	0.01
GLY3	Total	9,915,965	5,677,422	8527	**550,744**	474,821	12,345	3,247,324	426,107	89,633	4243	1279	29,482
	% of Total	100.00	57.26	0.09	**5.55**	4.79	0.12	32.75	4.30	0.90	0.04	0.01	0.30
	uniq	1,472,793	870,393	4467	**13,287**	8952	246	576,405	10,643	1523	211	76	834
	% of uniq	100.00	59.10	0.30	**0.90**	0.61	0.02	39.14	0.11	0.02	0.00	0.00	0.01

3ADT&length filter: reads removed due to 3ADT not found and length with < 18 nt and > 25 nt were removed (for plants); length with< 18 and > 26 were remove (for animals); Junk reads: Junk: > = 2 N, > = 7A, > = 8C, > = 6G, > = 7 T, > = 10Dimer, > = 6Trimer, or > =5Tetramer; Rfam: Collection of many common non-coding RNA families except micro RNA; Repeats:Prototypic sequences representing repetitive DNA from different eukaryotic species

Next, identification of the known miRNAs and novel miRNAs was accomplished by mapping the unique sequences in miRBase 20. The results showed that some miRNAs had a common precursor (pre-miRNA), for example, osa-MIR159f-p5 and osa-miR159f_R-1. In all, 479 unique miRNAs were detected in this study, and approximately 41.96% of the detected miRNAs were 21 nt in length, followed by 24 nt (29.23%) and 22 nt (14.61%) in length (Supplementary Table S6). There were 385 known miRNAs from 59 families from corresponding pre-miRNAs, and 94 miRNAs were identified as novel in this study (Supplementary Table S7). A total of 283 and 218 common miRNAs were detected in the three repeats of CK and GLY samples, respectively, of which 81 miRNAs were CK-specific, 16 miRNAs were GLY-specific, and 202 miRNAs were common to both, CK and GLY samples (Fig. 4b, Supplementary Table S8).

After differential analysis, 52 miRNAs were identified with differential expression (*P* < 0.05) in GLY and CK samples, of which 37 were known rice miRNA, including 21 down-regulated miRNAs and 16 up-regulated miRNAs (Fig. 4c, Supplementary Table S9). Of these differentially expressed miRNAs (DEMs), the expression of osa-MIR167f-p3 increased the most (5.76-fold), while that of osa-miR169r-3p decreased the most (4-fold).

### Degradome sequencing data and target analysis

Degradome sequencing was conducted to explore the targeted mRNAs of the conserved miRNAs and novel miRNAs. In total, degradome sequencing generated 12,455,265 raw reads. A total of 1,970,447 unique raw reads were obtained after the removal of low-quality and repetitive sequences. These reads were mapped to the rice transcriptome, and 9668,203 (approximately 77.62%) reads were mapped to the reference data (Table 4), followed by their candidate target gene identification. A total of 3547 targets (transcripts) were predicted for 317 conserved miRNAs and 58 novel miRNAs. After screening at a *P* value < 0.05, 268 targets (transcripts) from 64 conserved miRNAs were obtained, of which 216 targets (transcripts) were identified from 48 known rice miRNAs. Notably, a total of 32 targets (gene symbol) were identified from the degradome with *P* < 0.05 (Supplementary Table S10).

**Table 4:** Summary of degradome sequencing data

Sample	Number	ratio
Raw Reads	12,455,265	/
reads < 15 nt after removing 3 adaptor	42,877	0.34%
Mappable Reads	12,412,388	99.66%
Unique Raw Reads	1,970,447	/
Unique reads < 15 nt after removing 3 adaptor	13,762	0.70%
Unique Mappable Reads	1,956,685	99.30%
Transcript Mapped Reads	9,668,203	77.62%
Unique Transcript Mapped Reads	1,420,138	72.07%
Number of input Transcript	52,424	/
Number of Coverd Transcript	32,666	62.31%

**Fig. 5:**
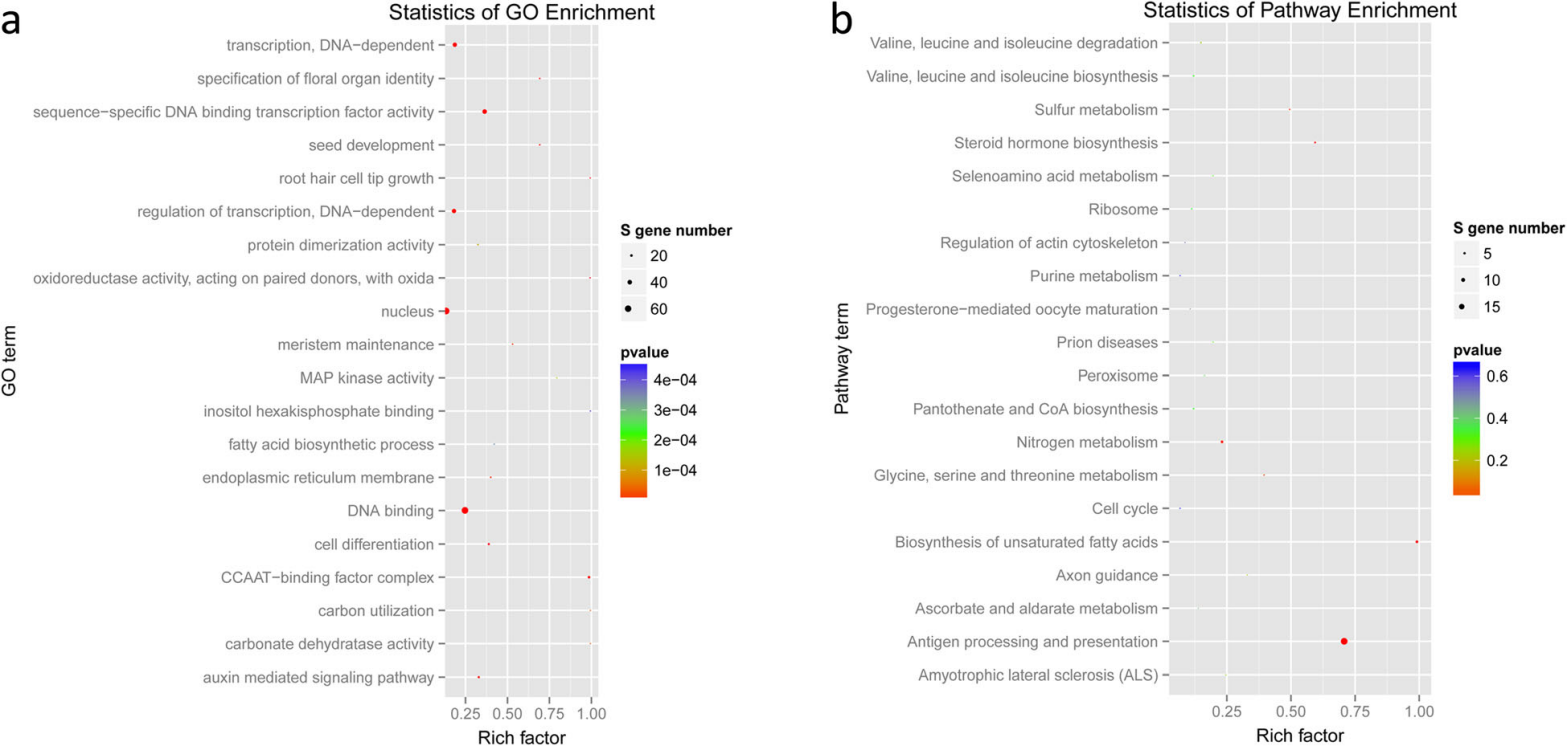
The results of functional enrichment analysis of the targets of miRNA identified through degradome sequencing analysis. **a** part of the significantly enriched GO terms. **b** part of the significantly enriched KEGG pathways

Target genes were notably enriched in various GO terms and KEGG pathways, such as ko04612~antigen processing and presentation, GO:0016602~CCAAT-binding factor complex; GO:0009734~auxin-mediated signaling pathway, GO:0006355~regulation of transcription, DNA-dependent, and GO:0006351~transcription, DNA-dependent, etc. (Fig. 5). The results were partially consistent with the significantly enriched GO terms and KEGG pathways for DEGs and DELSs, for instance, DNA-dependent transcription-related GO_BP terms.

### LncRNA-miRNA-mRNA regulatory network

**Fig. 6:**
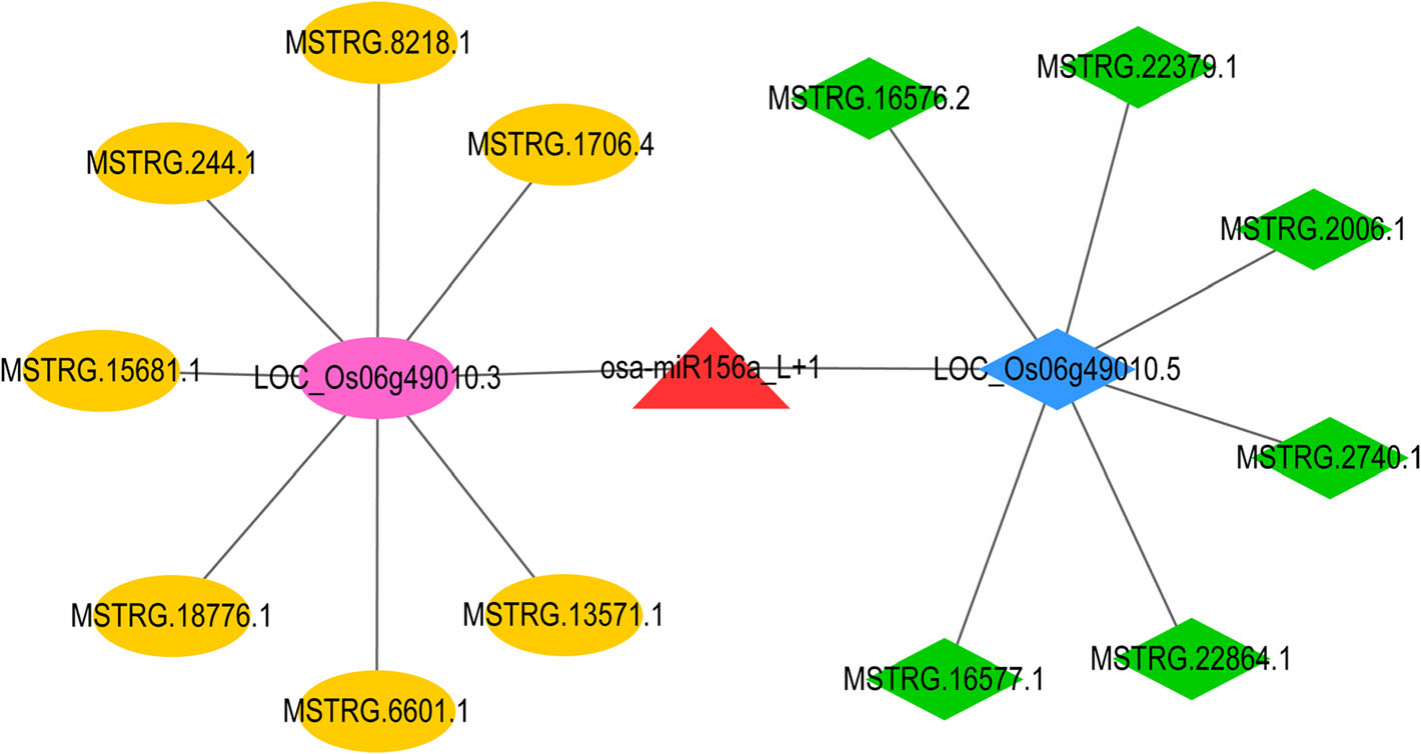
The lncRNA-miRNA-mRNA regulatory network. Red triangle represents up-regulated miRNAs; yellow circle represents up-regulated lncRNA, red circle represents up-regulated mRNA, green prismatic shape represents down-regulated lncRNA, and blue prismatic shape represents down-regulated mRNA

Analysis of sequencing data revealed the presence of 1197 DEGs, 131 DELs, 52 DEMs, in addition to 94 novel miRNAs identified in the GLY samples when compared to CK samples. This indicates changes due to glyphosate treatment led to visible transcriptome and degradome changes. To further explore their potential regulatory mechanism, an integrated analysis of lncRNA-miRNA-mRNA regulatory network was performed. First, the co-expression analysis between DEGs and DELs was conducted according to the methods described above, and a total of 3759 positively correlated lncRNA-mRNA co-expression pairs were obtained at the threshold of correlation coefficient r > 0.95 and *P* < 0.001 (Supplementary Table S11). The miRNA-mRNA interaction pairs were obtained based on the miRNA-targets identified from the degradome sequencing data and target prediction. The lncRNA-miRNA-mRNA regulatory pairs were further integrated based on the common mRNA of lncRNA-mRNA co-expression pairs and miRNA-mRNA interactions pairs, followed by visualization of lncRNA-miRNA-mRNA regulatory network using Cytoscape, an open source bioinformatics software. As shown in Fig. 6, the lncRNA-miRNA-mRNA regulatory network contained osa-miR156a_L + 1, squamosa promoter-binding-like protein 12 (*SPL12*, *LOC_Os06g49010.3* and *LOC_Os06g49010.5*), and 13 lncRNAs (eg., MSTRG.244.1 and MSTRG.16577.1). Of which, *SPL12* was enriched in several GO terms, including GO:0003677~DNA binding, GO:0005634~nucleus, GO:0006351~transcription, DNA-dependent, GO:0006355 ~regulation of transcription, DNA-dependent, and GO:0046872 ~metal ion binding.

### Expression level of mRNAs, miRNAs and lncRNAs determined by qPCR

**Fig. 7:**
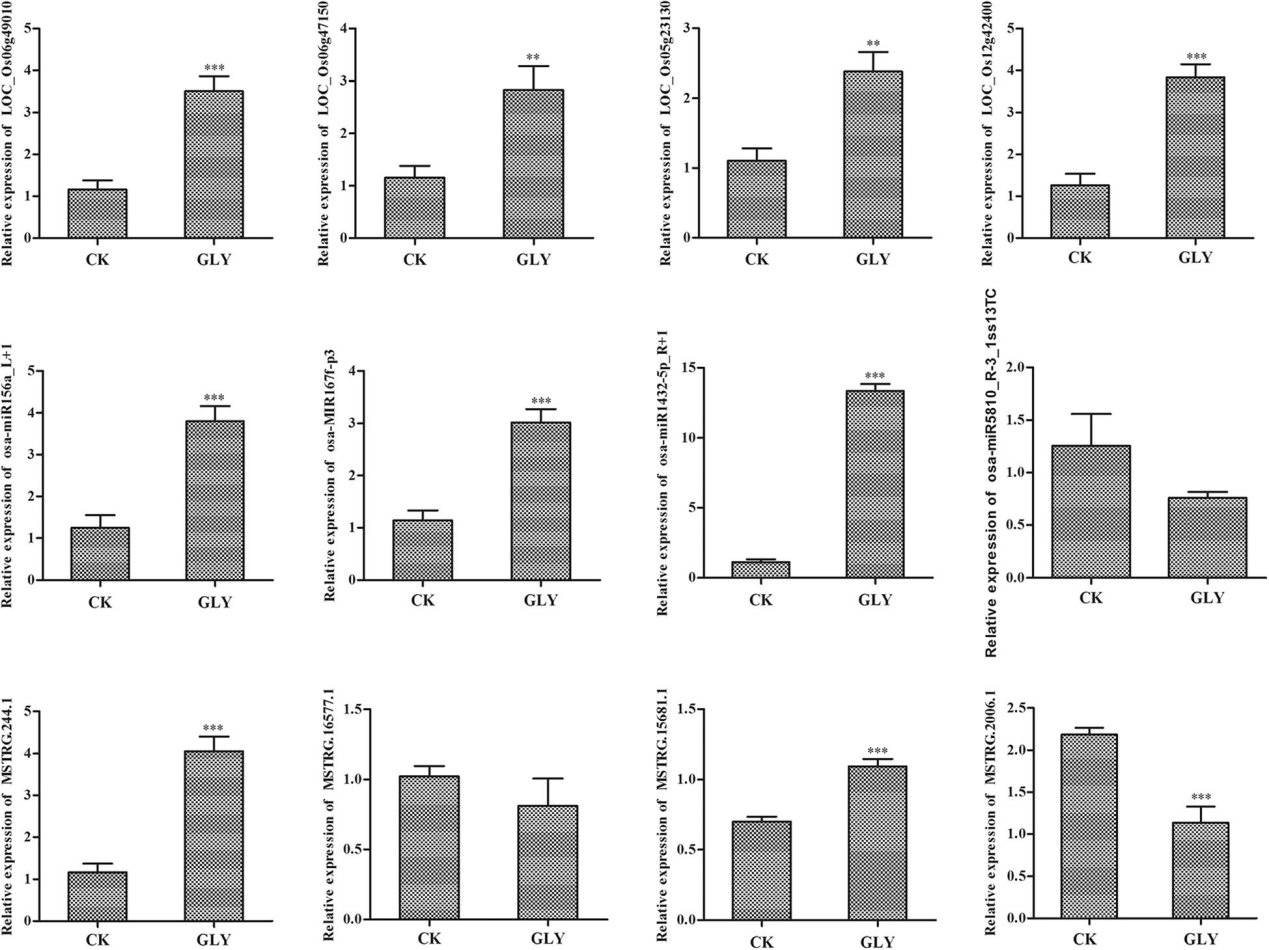
Expression level of key mRNAs, miRNAs and lncRNAs determined by qPCR. **P < 0.01; ***P < 0.001

Based on the results of sequencing analysis, the expression of several mRNAs, lncRNAs and miRNAs were verified in CK samples and GLY samples (Fig. 7). Expression trends were consistent for mRNAs, miRNAs and lncRNAs in both sequencing and qPCR analyses. The expression of osa-miR156a_L + 1 (the only miRNA in ceRNA network) showed a higher level in GLY samples. In addition, the expression of osa-MIR167f-p3, osa-miR1432-5p_R + 1 and osa-miR5810_R-3_1ss13TC were also determined. Of which, osa-MIR167f-p3 and osa-miR1432-5p_R + 1 were significantly up-regulated in GLY samples, while no significant difference was determined for the expression of osa-miR5810_R-3_1ss13TC, showing a decreasing tendency. The expression level of *SPL12* (*LOC_Os06g49010*), *NFYA10* (*LOC_Os12g42400*), *ARF18* (*LOC_Os06g47150*), and *LOC_Os05g23130* were up-regulated in GLY samples. Of which, *SPL12* (*LOC_Os06g49010*) was the gene in ceRNA network, and *LOC_Os05g23130* was a DEG with large fold changes in sequencing analysis, while *NFYA10* (*LOC_Os12g42400*), *ARF18* (*LOC_Os06g47150*) were the genes from degradome sequencing. Moreover, the expression of four lncRNAs in ceRNA network were determined, including MSTRG.244.1, MSTRG.15681.1, MSTRG.16577.1, and MSTRG.2006.1. Of which, there was no significant difference for the expression of MSTRG.16577.1, showing a decreasing tendency. The expression of the other three lncRNAs were consistent with sequencing analysis.

## Discussion

To better understand the global gene expression and the potential regulatory mechanisms involved in the adaptive responses to glyphosate application in rice, systematic and integrated analyses of transcriptome and degradome based on high-throughput sequencing technology were performed. In this study, 1197 genes, which included 598 up-regulated genes and 599 down-regulated genes, showed significantly different expression in the leaves of GLY compared to that of CK. These genes were found to be implicated in various biological processes, including peroxidase activity, glutathione metabolic, hormone-mediated signaling pathway, which were the biological processes involved in the detoxification of plant response to stress factors, such as drought, salinity and temperature [29, 30]. Peroxidases are involved in various physiological processes, for example, abiotic stresses. They function by mediating cell elongation to control cell growth, and they play crucial roles in the synthesis and metabolism of auxin, ethylene, flavonoids and secondary metabolites [31, 32]. In plants, the roles of glutathione peroxidases in detoxification has been demonstrated in many species and under different stress factors [32, 33]. Ahsan et al. using proteomic approach, demonstrated that oxidative stress was generated with glyphosate application in rice. Antioxidant enzymes (glutathione S-transferase, peroxidases and so on) accumulated to help protect the plants from the adverse stress of glyphosate [34]. Chlorophyll content is one of the important indices of physiological metabolism in plants. Glyphosate inhibits the synthesis of aromatic amino acids by inhibiting the shikimic acid pathway, leading to reduced chlorophyll content in plants [35, 36] [37]. In the present study, only the up-regulated DEG *LOC_Os12g34874* (*LOC_Os12g34874.1*; *LOC_Os12g34874.2*; *LOC_Os12g34874.3*) were implicated in both GO:0009073~aromatic amino acid family biosynthetic processes and GO:0009570~chloroplast stroma. The most famous glyphosate stress-responsive gene *EPSPS* (*LOC_Os06g04280*) was also detected in this study. Although the expression of *LOC_Os06g04280.1* showed an increased trend (1.00-fold) in GLY samples than that in CK samples, it was not a significant DEG based on the preset significance threshold, which indicated that there might be other significant genes in response to glyphosate stress.

Recent advances in plant miRNA research have been possible due to advancements in high-throughput sequencing. Plant miRNAs play crucial roles in biotic and abiotic stress responses, including in drought [38] and salt stress [39]. In the present study, 385 known miRNAs from 59 miRNA families and 94 novel miRNAs were identified from GLY and CK samples, and most of them were 21 nt in length, followed by 22 nt and 24 nt. In addition, the degradome sequencing analysis was used to identify miRNA targets, and this technology has been widely used in many plants. One degradome library containing all the six samples was constructed, and 32 target genes were subsequently identified, for example, *SPL12* was identified as a target of osa-miR156a_L + 1.

miR156 is a highly conserved member of the miRNA family in plants, and is involved in regulating plant development [40]. Changes to plant morphology were observed when miR156 was over-expressed in Arabidopsis [41], rice [42] and maize [43], indicating its role in plant development. Cui et al. reported that miR156 was induced to maintain its juvenile state for a long time under stress, and that miR156 was inhibited under favorable conditions to accelerate the development transition [44]. In addition, plant sensitivity to stress increased when the miR156 signaling pathway was blocked, which was reversed when miR156 was over-expressed in Arabidopsis and rice. This effect of miR156 was found to be related to the miR156-SPL9-DFR pathway [44]. Yin et al. revealed that miR156/SPL9 mediated an immune response through the regulation of ROS accumulation and the activation of salicylic acid (SA) signaling in *Arabidopsis thaliana* [45]. Ge et al. showed negative regulation of miR156 to brown planthopper tolerance in rice, mainly by decreasing the jasmonic acid (JA) signaling. The authors suggested that miR156-SPL pathway might be involved in regulating JA biosynthesis [46]. The SPL (squamosa promoter-binding like) family of transcription factors are plant-specific and share a highly conserved DNA-binding domain of approximately 78 amino acid residues, which affect a broad range of plant physiology and developmental events, including embryogenesis, shoot and leaf development, and flowering [47, 48]. A total of 19 *SPL* genes divided into six subgroups were identified in rice [49]. Currently, six rice *SPLs* (*OsSPLs*) have been identified, including *OsSPL6*, *OsSPL8*, *OsSPL13*, *OsSPL14*, *OsSPL16*, and *OsSPL12*, which are implicated in rice grain size and shape (*OsSPL13* and *OsSPL16*) [50, 51], panicle branching and plant architecture (*OsSPL6*, *OsSPL8* and *OsSPL14*) [52–54], and crown root development (*OsSPL12* and *OsSPL13*) [55]. To the best of our knowledge, there are no reports on the role of miR156-*SPL12* in rice in response to glyphosate. Hence, we speculate that the regulation of *OsSPL12* by OsmiR156 might be associated with response generation to glyphosate application. Several lncRNAs (eg., MSTRG.244.1 and MSTRG.16577.1) and *SPL12* interact through competition with osa-miR156 based on the lncRNA-miRNA-mRNA regulatory network. Despite the function of lncRNAs has just begun to be recognized in plants, plant lncRNAs are reportedly involved in photomorphogenesis, auxin transport and flowering [24, 25]. For instance, lncRNAs COLDAIR and COOLAIR mediate flowering time, probably by regulating the FLOWERING LOCUS C (*FLC*) in Arabidopsis [26, 27]. Therefore, we suggest the presence of a potential ceRNA mechanism in response to glyphosate stress in rice involving osa-miR156, lncRNAs (e.g., MSTRG.244.1 and MSTRG.16577.1) and *SPL12*. In the current study, *SPL12* was enriched in DNA binding, regulation of transcription, and metal ion binding. Kang et al. suggested that various signaling pathways were implicated in leaf growth and development in response to different stress, including photosynthesis, signal transduction, transcription and so on [56]. Metal ion concentration in tissues may affect the presence and transfer of glyphosate in tissues due to the chelating nature of glyphosate [57]. Studies had showed that the formation of metal−glyphosate complexes could affect the herbicidal effect of glyphosate [58]. Yang et al. uncovered that *ApY2SK2* and *ApSK3* dehydrin genes could enhance plant stress tolerance probably by regulating ROS metabolism and metal ions binding [59]. Hence, we speculate that MSTRG.244.1/ MSTRG.16577.1- osa-miR156- *SPL12* might take part in the responses of rice to glyphosate stress probably by regulating transcription and metal ions binding. Despite of the novel findings, there were still some limitations in the current study. Our study preliminarily analyzed and determined the expression of several mRNAs, miRNAs and lncRNAs, and predicted potential ceRNA axis. Thus, further experiments, including quantitative PCR, dual luciferase reporter assay, as well as functional experiment are needed to validate the expression of more genes, regulatory relationships and their possible roles in response to glyphosate-stress in rice.

## Conclusion

To the best of our knowledge, this is the first study to report on an integrated analysis of responses of mRNAs, lncRNAs, and miRNAs to glyphosate-stressin rice. We identified several lncRNAs (e.g., MSTRG.244.1 and MSTRG.16577.1) and *SPL12* that interact through competition for osa-miR156, which might be a potential ceRNA mechanism in response to glyphosate stress in rice by regulating transcription and metal ions binding. These findings mayprovide a theoretical basis for breeding glyphosate-tolerant rice varieties and for further research on the biogenesis of glyphosate-tolerance in rice.

## Methods

### Plant materials and treatment

The glyphosate-resistant rice CA21 and the glyphosate-sensitive rice P1003 were used in this research work. The glyphosate-tolerant rice CA21 was developed by Institute of Crop and Nuclear Technology Utilization, Zhejiang Academy of Agricultural Sciences, which was obtained by mutagenizing of Zhejing30 with 350 Gy 60Co-γ radiation and with 0.5–1% ethyl methane sulfonate (EMS) and then multi-generation hybridizing and backcrossing with P1003. The glyphosate-sensitive rice P1003 was obtained from RiceTec Inc. They were planted in 60 × 30 cm bowls. Glyphosate (100% glyphosate powder, Zhejiang Xin ‘an Chemical Group Co., Ltd.) with an effective spray dose of 2 mg·ml^− 1^ to treat the rice seedlings at the third-leaf stage. Once all the P1003 plants died, the leaves from CA21 that were sprayed with 2 mg ml^− 1^ glyphosate (GLY) and those plants without any treatment (CK) were collected for high-throughput sequencing analysis. The experiments were conducted in the greenhouse of the Institute of Crop and Nuclear Technology Utilization, Zhejiang Academy of Agricultural Sciences in November 2017. Three replications were maintained for each group.

### Determination of physiological indicators

Leaves of the CA21 and P1003 were collected on the third day after glyphosate treatment, and were used in the determination of shikimic acid, malondialdehyde (MDA), and glutathione transferases (GSTs) levels. The standard curve of shikimic acid was developed using the method described by Ye et al. [28]. Light absorption was determined at 380 nm using a 756 UV-Visible spectrophotometer (Shanghai Lingguang Technology Instrument Co. Ltd.), followed by the estimation of shikimic acid content based on the standard curve. MDA and GST levels were determined using the MDA kit (Suzhou Keming Biotechnology Co. Ltd) and GST assay kit (Suzhou Keming Biotechnology Co. Ltd), and following manufacturer instructions. Three replications were maintained and the mean value was used to represent the final levels of each physiological indicator.

### RNA isolation

TRIzol® reagent (Invitrogen) was used for Total RNA extraction from each sample. RNA quality and purity were determined by using the Bioanalyzer 2100 and RNA 6000 Nano LabChip Kit (Agilent, CA, USA) with a RIN number > 7.

### cDNA library construction, sequencing and analysis

Ribosomal RNA was depleted as per the instructions provided in the Epicenter Ribo-Zero Gold Kit (Illumina, San Diego, USA). The cDNA libraries were created following fragmentation of the poly(A)−/(A) + RNA fractions, reverse-transcription. Paired-end sequencing was conducted on an Illumina HiSeq 4000 (LC-Bio, China). The reads with adaptor contamination, low quality and undetermined bases were removed, followed by quality verification using FastQC (http://www.bioinformatics.babraham.ac.uk/projects/fastqc/). We used Bowtie 2 [60] and TopHat2 [61] to map reads to the genome of Nipponbare rice Reference Genome (IRGSP build 5.0). The mapped reads of each sample were assembled using StringTie [62]. After the final transcriptome was generated, Ballgown [63] was used to estimate the expression levels of all transcripts. The raw data was available at NCBI Gene Expression Omnibus (GEO) repository with Accession Number GSE142323.

### LncRNA identification

Transcripts that overlapped with known mRNAs and transcripts shorter than 200 bp were discarded. Long non-coding RNA (lncRNA) prediction was performed for the remaining transcripts using CPC [64] and CNCI [65], which are used to predict transcripts with coding potential. All transcripts with CPC score < − 1 and CNCI score < 0 were removed. The remaining transcripts were considered as lncRNAs.

### miRNA identification

sRNAs were ligated to 3′- and 5′-adaptors using T4 RNA ligase. The construction of sRNA library was completed using approximately 1 μg total RNA and following the protocol of TruSeq Small RNA Sample Prep Kit (Illumina, San Diego, USA), and single-end sequencing (36 bp) on an Illumina HiSeq 2500 (LC-Bio, China). Raw data were processed to remove the adaptor sequences, and short sequences with base length less than 18 nt were eliminated. Sequences with low complexity, and those remaining were matched to ribosome RNA (rRNA), transport RNA (tRNA), small nucleolar RNA (snoRNA), small nuclear RNA (snRNA), as well as other repetitive sequences using ACGT101-miR (LC Sciences, Houston, Texas, USA). The known miRNAs and novel miRNAs were identified by mapping the unique sequences to specific species precursors in miRBase 20.0 (http://www.mirbase.org/). The raw data of miRNA-Seq was available at NCBI GEO repository with Accession Number GSE142110.

### Degradome library construction, sequencing and analysis

The degradome library was constructed using approximately 20 μg of total RNA according to a previously described method [66], with several modifications. The poly(A) + RNA was mixed and annealed using biotinylated random primers. RNAs with 5′-monophosphates were ligated to 5′ adapters. Libraries were single-end sequenced (36 bp) using the 5′ adapter only on an Illumina HiSeq 2500 (LC-Bio, China). The sequencing data was analyzed using CleaveLand3.0 [67]. The degradome reads were matched to transcriptome to generate the degradome density file using script “CleaveLand3_map2dd.pl” [67]. In addition, the targeted mRNA sequences of the miRNAs were predicted using TargetFinder software [68]. The overlapped mRNAs of the predicted target mRNAs and the mRNAs in degradome density file were considered as the target genes of the miRNAs. The raw data of degradome-Seq was available at NCBI GEO repository with Accession Number GSE142265.

### Differential expression and functional enrichment analyses

In order to compare the expression levels of mRNA, lncRNA and miRNA between GLY and CK, the DESeq in R package was used [69]. Differentially expressed mRNAs/lncRNAs/miRNAs were screened with a threshold value of |log fold change (FC)| ≥ 1, and *P* value < 0.05. Besides, a hypergeometric test in clusterProfiler (version:3.8.1, http://bioconductor.org/packages/release/bioc/html/clusterProfiler.html) was performed to assess enrichment of the Gene ontology (GO) terms and KEGG pathways in differentially expressed mRNAs or the target genes of lncRNA/miRNA. We selected the terms with *P* value < 0.05 as significantly enriched.

### Integrated analysis

Pearson correlation coefficient of lncRNA and mRNA were calculated using the corresponding matrix data, and followed by a correlation test. According to the competing endogenous RNAs (ceRNA) mechanism, significant positively correlated co-expression of lncRNA-mRNA were screened at a correlation coefficient r > 0.95 and *P* value < 0.001. In addition, the significantly differentially expressed miRNA-mRNA interactions in the degradome, and the differentially expressed miRNAs in the sRNA library were screened. The lncRNA-miRNA-mRNA interactions were integrated based on the miRNA-mRNA pairs in the degradome, and the differentially expressed miRNAs in the sRNA library (*P* < 0.1, this threshold value was selected to better integrate the data from the transcriptome, sRNA, and the degradome) and the co-expressed lncRNA-mRNA pairs. The lncRNA-miRNA-mRNA regulatory network was visualized using Cytoscape [70] (version 3.4.0, http://chianti.ucsd.edu/cytoscape-3.4.0/).

### Quantitative PCR (qPCR)

Based on the results of sequencing analysis, the expression of several mRNAs, lncRNAs and miRNAs were verified in CK samples and GLY samples. Total RNA was extracted using TRIzol reagent (Invitrogen, Carlsbad, CA, USA) following the manufacturer’s instructions. The optical absorbance ratio at 260/280 nm was measured using Scandrop 100 (Analytic Jena, Germany) to determine the concentration and quality of the RNA. Complementary DNA (cDNA) was synthesized was generated using a TUREscript First-Strand cDNA Synthesis Kit (Aidlab, China). Real-time qPCR was performed to validate gene expression using 2 × SYBR® Green Supermix on an analytikjena-qTOWER2.2 PCR System (Analytik Jena, Germany) with the following thermal cycling conditions: 95 °C for 3 min, followed by 39 cycles at 95 °C for 10 s and 60 °C for 30 s. The melting curve was analyzed from 60 °C to 95 °C at an increment of 1 °C/cycle, holding time 4 s. The specific primer pairs of the validated genes are shown in Supplementary Table S12. The relative quantification of gene expression was based on the comparative CT (2 − ^ΔΔCT^) method [71]. Statistical analysis was performed using GraphPad Prism 5 (GraphPad Software, San Diego, CA, USA). Data were presented as mean ± standard deviation (SD), with a statistical significance of *P* < 0.05.

## Supplementary information

**Additional file 1 : Figure S1** The distribution of the unique mapped reads.

**Additional file 2 : Figure S2** Types and proportions of lncRNAs.

**Additional file 3 : Table S1** Sequence read statistics based on alignment to a Reference Genome.

**Additional file 4 : Table S2** Statistics of gene expression values for each sample.

**Additional file 5 : Table S3** Statistics of novel lncRNAs in each sample.

**Additional file 6 : Table S4** Statistics of differentially expressed lncRNAs.

**Additional file 7 : Table S5** mRNA-lncRNA interaction analysis.

**Additional file 8 : Table S6** Length distribution of unique miRNAs.

**Additional file 9 : Table S7** Summary of known and predicted miRNAs.

**Additional file 10 : Table S8** Venn analysis of detected miRNAs.

**Additional file 11 : Table S9** Statistics of differentially expressed miRNAs.

**Additional file 12 : Table S10** Significant degradome results.

**Additional file 13 : Table S11** Positively correlated lncRNA-mRNA co-expression pair.

**Additional file 14 Table S12** Specific primer pairs of the validated mRNAs, miRNAs and lncRNAs.

## Data Availability

The raw data for samples used in this analysis have been deposited in the NCBI Gene Expression Omnibus (GEO) repository with Accession Number GSE142323, GSE142266 (subseries GSE142110 and subseries GSE142265). The data in GEO repository are scheduled to be publicly-available on: Dec, 2022. The data are also available at https://pan.baidu.com/s/1Lvni1J3bkjejFWEau9HYbw (transcriptome-seq) and https://pan.baidu.com/s/1PToyC1JDn0uY6mvHW0tY9g (degradome-seq;miRNA-Seq), and the extraction code can be obtained from the corresponding author upon reasonable request.

## Abbreviations

ARF18: Auxin response factor 18
BP: Biological process
CC: Cellular component
cDNA: Complementary DNA
ceRNA: Competing endogenous RNAs
CK: CA21 plants with no spray
DEGs: Differentially expressed mRNAs
DELs: Differentially expressed lncRNAs
DEMs: Differentially expressed miRNAs
EMS: Ethyl methane sulfonate
EPSPS: 5-enolpyruvylshikimate-3-phosphate synthase
FLC: FLOWERING LOCUS C
GAT: Lyphosate acetyltransferase
GEO: Gene expression omnibus
GLY: CA21 sprayed with 2 mg·ml^− 1^ glyphosate
GO: Gene ontology
GOX: Oxidoreductase
GSTs: Glutathione transferases
JA: Jasmonic acid
LncRNAs: Long ncRNAs
MDA: Malondialdehyde
MF: Molecular function
ncRNA: Non-coding RNAs
NFYA10: Nuclear factor Y, subunit A10
qPCR: Quantitative polymerase chain reaction
rRNA: Ribosome RNA
SD: Standard deviation
snoRNA: Small nucleolar RNA
snRNA: Small nuclear RNA
SPL: Squamosa promoter-binding-like protein
sRNA: Small RNA
tRNA: Transport RNA
