# Supplementary figures and images for: Identification and integrated analysis of glyphosate stress-responsive microRNAs, lncRNAs, and mRNAs in rice using genome-wide high-throughput sequencing
Source: BMC Genomics. 2020 Mar 17;21:238. doi: 10.1186/s12864-020-6637-6 (PMC7076996; doi:10.1186/s12864-020-6637-6)

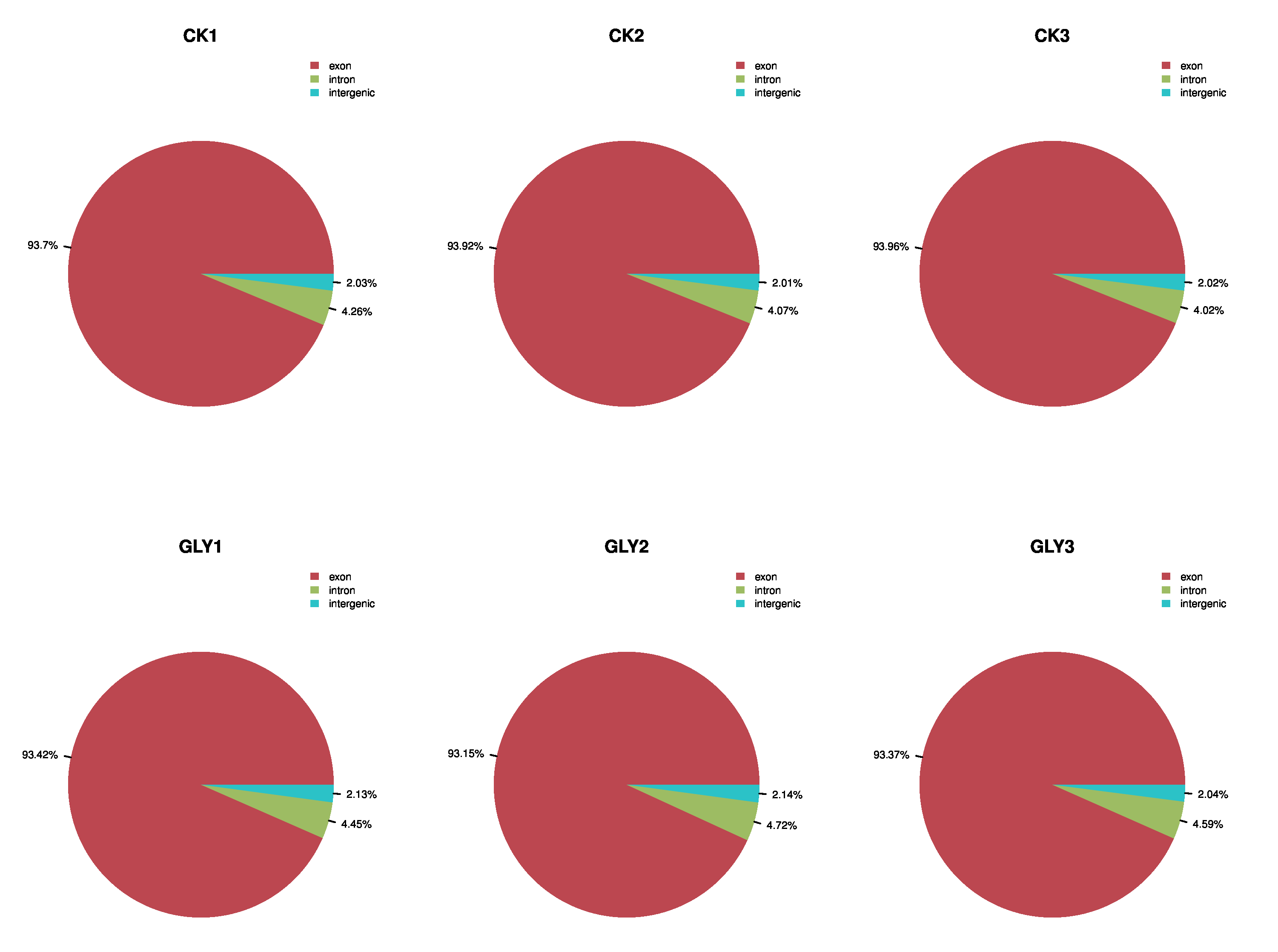

Supplement: Supplementary file 1 — Additional file 1 : Figure S1 The distribution of the unique mapped reads. [file 12864_2020_6637_MOESM1_ESM.tif]

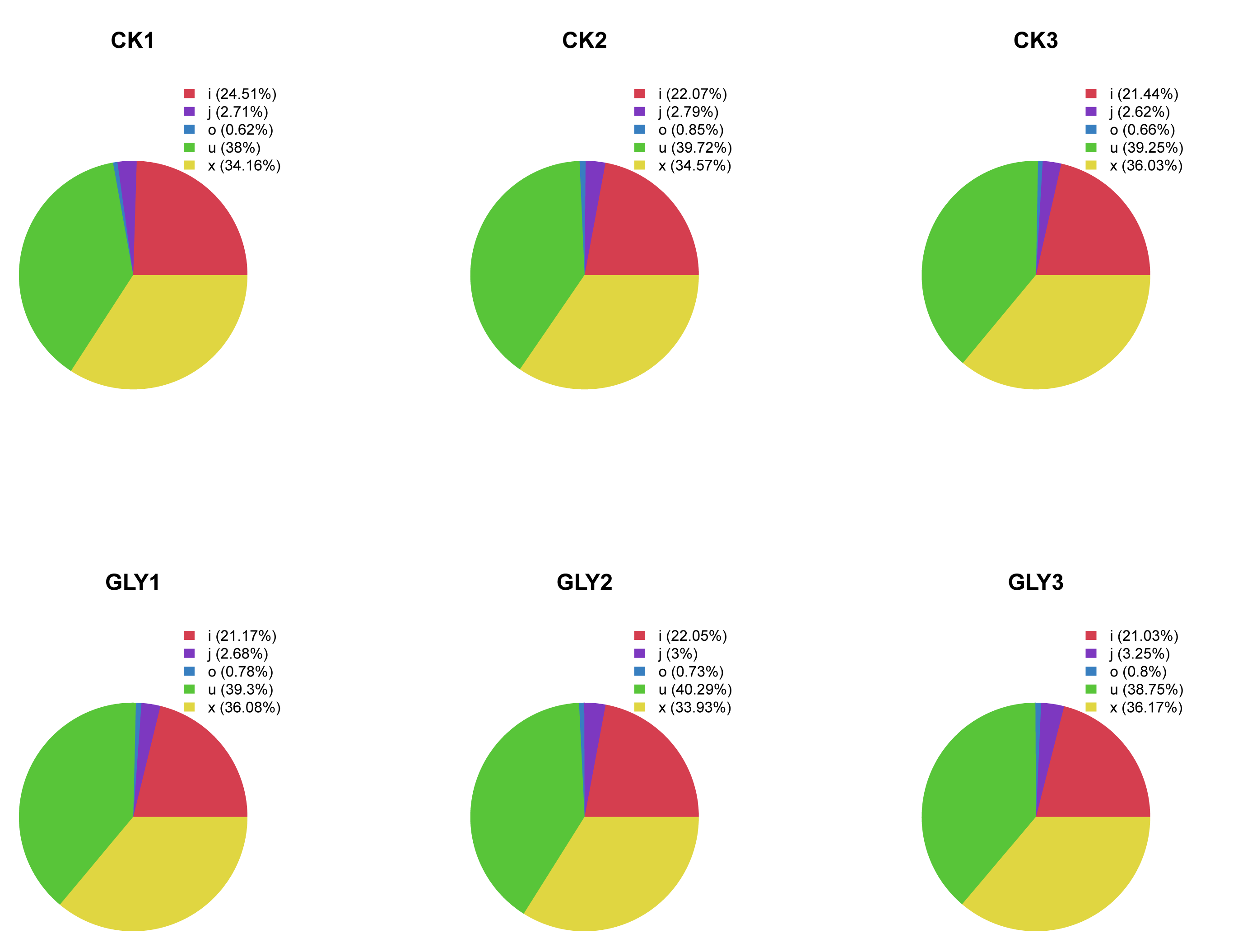

Supplement: Supplementary file 2 — Additional file 2 : Figure S2 Types and proportions of lncRNAs. [file 12864_2020_6637_MOESM2_ESM.tif]
